# Metagenomes from the Saline Desert of Kutch

**DOI:** 10.1128/genomeA.00439-14

**Published:** 2014-05-15

**Authors:** A. S. Pandit, M. N. Joshi, P. Bhargava, G. N. Ayachit, I. M. Shaikh, Z. M. Saiyed, A. K. Saxena, S. B. Bagatharia

**Affiliations:** Gujarat State Biotechnology Mission, Department of Science and Technology, Government of Gujarat, Gandhinagar, Gujarat, India

## Abstract

We provide the first report on the metagenomic approach for unveiling the microbial diversity in the saline desert of Kutch. High-throughput metagenomic sequencing of environmental DNA isolated from soil collected from seven locations in Kutch was performed on an Ion Torrent platform.

## GENOME ANNOUNCEMENT

Studies of hypersaline ecosystems have improved our understanding of the biology of extreme environments, and many have resulted in the discovery of novel organisms and enzymes with enhanced potential for biotechnological applications (1–3). One of the unique and unexplored hypersaline ecosystems in India is the Great Rann of Kutch, a saline desert located in Gujarat, India. Stretching over an area of 7,505 square kilometers, it is one of the largest salt deserts in the world (4). Despite the possibility of the presence of novel microbes with high economic and industrial potential, there are no detailed reports on the microbial diversity of this region.

To study the influence of salinity on microbial communities, we collected soil samples from 7 locations (S1 [23°48′39″N, 70°58′60″E], S2 [23°47′33″N, 71°0′29″E], S3 [23°54′29″N, 70°32′16″E], S4 [23°56′29″N, 70°11′18″E], S5 [23°50′6″N, 69°31′8″E], S6 [23°56′27″N, 70°11′18″E], and S7 [23°56′26″N, 70°11′16″E]) along a transect of 150 km with different salinity levels. Electrical conductivity and pH of the soils ranged from 68.5 mS/cm to 513.3 mS/cm and 7.2 to 8.6, respectively. Community DNA was extracted using a Power Max soil DNA isolation kit (MO BIO Laboratories, Inc., Carlsbad, CA, USA). High-throughput sequencing of metagenomic DNA was done using Ion Express Template 300 chemistry on an Ion Torrent Personal Genome Machine. Filtered reads containing 16S rRNA gene sequences were identified by a BLASTN search against NCBI 16S rRNA sequences (*Bacteria* and *Archaea*) with an E value of 1e−5. Taxonomic affiliations were assigned using Ribosomal Database Project RDP MultiClassifier 1.1 (5) with a 50% bootstrap confidence cutoff. Raw sequence reads of all seven data sets were also submitted to the MG-RAST v. 3.3.7.3 (6) and IMG/M (7) databases for downstream analysis. Within MG-RAST, taxonomic identification was based on the top BLAST hit to the M5NR database, with an E value cutoff of 1e−5 and 60% identity.

Using a shotgun metagenomic approach, we obtained a total of 1.6 to 3.1 million sequences per sample with a read length of 100 to 159 bp. The percentages of GC content of the metagenomes were high and ranged from 51 to 60%. Metagenomes on average were constituted of 56 to 87% bacteria and 8 to 40% archaea. Major prokaryotic phyla observed were *Proteobacteria* (abundance percentages of 18.6 to 47.7%), *Euryarchaeota* (7.9 to 40.1%), *Bacteroidetes* (9.1 to 18.7%), *Firmicutes* (5.8 to 8.4%), *Actinobacteria* (2.1 to 5.8%), and *Cyanobacteria* (2 to 4.2%).

This is the first report on the metagenomics of the saline desert of Kutch. It adds data on the composition as well as survival strategies of microbial communities in a saline desert. This study will also create a basis for building microbial exploration strategies for this special ecogeographic niche.

### Nucleotide sequence accession numbers.

The DNA sequences from this metagenomic project have been deposited in the NCBI Sequence Read Archive under the accession numbers SRX306504, SRX306503, SRX519631, SRX519746, SRX519747, SRX519748, and SRX519749.

## References

[1] Caton TM Witte LR Ngyuen HD Buchheim JA Buchheim MA Schneegurt MA 2004 Halotolerant aerobic heterotrophic bacteria from the Great Salt Plains of Oklahoma. Microb. Ecol. 48:449–462. 10.1007/s00248-004-0211-7 15696379

[2] Nicholson CA Fathepure BZ 2005 Aerobic biodegradation of benzene and toluene under hypersaline conditions at the Great Salt Plains, Oklahoma. FEMS Microbiol. Lett. 245:257–262. 10.1016/j.femsle.2005.03.014 15837380

[3] Ventosa A Mellado E Sanchez-Porrro C Marquez MC 2008 Halophilic and halotolerant microorganisms from soils, p 87–115 *In* Dion P Nautiyal CS (ed), Microbiology of extreme soils. Springer-Verlag, Berlin, Germany

[4] McGinley M 2008 Rann of Kutch seasonal salt marsh. *In* Encyclopedia of Earth. Environmental Information Coalition, National Council for Science and the Environment, Washington, DC http://www.eoearth.org/article/Rann_of_Kutch_seasonal_salt_marsh

[5] Wang Q Garrity GM Tiedje JM Cole JR 2007 Naive Bayesian classifier for rapid assignment of rRNA sequences into the new bacterial taxonomy. Appl. Environ. Microbiol. 73:5261–5267. 10.1128/AEM.00062-07 17586664 PMC1950982

[6] Meyer F Paarmann D D’Souza M Olson R Glass EM Kubal M Paczian T Rodriguez A Stevens R Wilke A Wilkening J Edwards RA 2008 The metagenomics RAST server—a public resource for the automatic phylogenetic and functional analysis of metagenomes. BMC Bioinformatics 9:386. 10.1186/1471-2105-9-386 18803844 PMC2563014

[7] Markowitz VM Chen IM Chu K Szeto E Palaniappan K Grechkin Y Ratner A Jacob B Pati A Huntemann M Liolios K Pagani I Anderson I Mavromatis K Ivanova NN Kyrpides NC 2012 IMG/M: the integrated metagenome data management and comparative analysis system. Nucleic Acids Res. 40:D123–D129. 10.1093/nar/gkr975 22086953 PMC3245048

